# Retraction: Activation of Nuclear Factor Kappa B (NF-kB) Signaling Pathway Through Exercise-Induced Simulated Dopamine Against Colon Cancer Cell Lines

**DOI:** 10.7759/cureus.r155

**Published:** 2024-12-20

**Authors:** Dhanushree Baranikumar, Meenakshi Sundaram Kishore Kumar, Venkataramanan Natarajan, Lavanya Prathap

**Affiliations:** 1 Department of Anatomy, Saveetha Dental College and Hospitals, Saveetha Institute of Medical and Technical Sciences, Saveetha University, Chennai, IND

This article has been retracted by the Editors-in-Chief due to the inclusion of multiple cited sources that do not correspond to the cited text or article topic. The authors did not respond to inquiries made by the journal. The journal greatly regrets that this was not identified prior to publication.

